# What We Most Need

**Published:** 1866-07

**Authors:** G. W. De Camp


### WHAT WE MOST NEED.

BY G. W. DE CAMP, D. D. S.

For centuries man seemed inclined to follow the mad experiment of living without "thought."

The reign of terror was but action void of "thought." France might have been blessed had the Jacobins been guided by reason. Was it not by close "thought' that Luther turned things upside down, and confounded the Pope? What was it that enabled Cadimus to grasp the keys with which he unlocked the storehouse of the immortal mind? How did Bacon show the fallacy of Grecian philosophy? How did Newton tell of the stars, and trace the law of gravitation in the falling apple? What nerved the hand of Franklin to grasp the thunderbolts from the armory of Heaven? Was it not "close thought?"

We want independent thought. "It has been said independent thinkers are few, and most Philosophers found their philosophy on the opinions of former Philosophers." "There is nothing new under the sun." Creation gives God as the author of all, but, though we may not claim originality among men, yet old thoughts may be clothed in new garbs, and beautified as the husbandman beautifies the earth. Not by relying on others for opinions, or by copying some writer, word for word; or by taking a sentence here and there, combining them, and calling it an "original communication;" nor yet, by taking other's thoughts, and imagine you can vend this second-hand furniture, and not be stamped a "Plagiarist."

We need singleness of thought. Not that man should have but one thought, and travel on in the same old way—like the good old Parson, of whom it is said, he preached fore-ordination from every text. Thoughts must be mastered one at a time, otherwise all will be lost. I think it was Franklin who told of a child who having an apple in each hand, tried to carry a third, and lost all. The old hen picks up grain by grain.

We need concentrated thought. The mind must be subject to the will, so that all the faculties may obey the call, rally to the rescue, grasp the subject, and not let go until the victory has been won. Small squads of soldiers may gain small victories, but it is only by a concentrated force that the lines are broken, and the enemy conquored. We need continued thought. What would a machine of great power and speed be worth, if it worked but at times? The patient who takes medicine to-day, and neglects it to-morrow, will surely come to grief. Tension breaks the bow, but thought strengthens the mind. It is by continued action that water wears the rock. As a fixed habit may become a necessary part of the existence, so may continued thought become a fixed habit. Then, instead of our finding thought a friendless outcast, we will be welcomed to her hospitalities, and find ourselves pleasantly entertained by her memberless friends.

One thought begets another. It is never found alone, but always in company with its relatives.

We need patient thought. Man has been defined as a religious animal, the laughing animal, the weeping animal, the whittling animal, and by Solomon's admonitions, by inference the lazy animal.

Many strong minds have given up the search for truth, on account of its tiresome paths, who might have blessed humanity, if they had nerved themselves to patient toil. By close thought, we purify our minds, and are able to see the little things which God has clothed in so much beauty. The farmer considers the Geologist a fool, as he looks at him carefully examining the little stone,—he could have shoveled a dozen cart-loads of them, or ploughed a half-dozen acres, in the same time. Close thought is needed, in order to impart instruction aright. True, some men's happiest efforts are made at an unexpected moment, but shall ordinary minds wait for poetic breathing?

What will follow if our thoughts are not independent, single, concentrated, continued, and patient; is to be seen by the present. The advantages of the day are great. No man can have a reasonable excuse for not being able to "act well his part." Colleges are not appreciated. They are valuable, but cannot supply the place of thought; but thought can, in some good degree, supply the place of colleges.

We need a higher standard of dental literature, if we expect to keep pace with the advance of science.

There are some, it would seem, who write simply that their names may appear in print. Their whole object is to let you know that *they* live. From their writings we are led to suppose that they oil the "*hub*" of the universe, and without their help things would come to a stand still, and like a "bug," as it flies through the air, cry "what a great dust we create."

We look to our journals as our hope. Let what is to be published, be written with thought—close thought. Let none use it as an instrument to injure his brother. Men, when they wish to write like fools, should write for "Artemus Ward's book." All such should be regular subscribers for "Ward" or "Nasby," but not contributors to a journal of the dental profession. They trail her robes in the mud. The times demand a higher grade of dental literature.

The wants of the profession demand it. We have been told of a vast unexplored field, until it has become an old and tiresome story. We need the charts. If these cannot be given, say and write no more until they can. Give us not fancied pictures of the future, but sketch the rude present.

Practical knowledge is our necessity. We need close thought *most*, because we have it *least*.

